# Study of Lipid-Modifying Therapy Use and Risk Factor Management in Patients With Dyslipidemia in Duhok City/Kurdistan Region, Iraq

**DOI:** 10.7759/cureus.53849

**Published:** 2024-02-08

**Authors:** Sipan Sarbast, Jamal B Mohamad

**Affiliations:** 1 Medicine, College of Medicine, University of Duhok, Duhok, IRQ

**Keywords:** obesity, risk factor, llt, cvd, ldl-c

## Abstract

Introduction

Atherosclerotic cardiovascular disease (ASCVD) is a leading cause of mortality globally, according to the World Health Organization. Research from the Middle East indicates that cardiovascular disease-related deaths in the region are among the highest worldwide. Multiple risk factors contribute to ASCVD. Elevated low-density lipoprotein cholesterol (LDL-C), often associated with hyperlipidemia, plays a pivotal role. The reduction of LDL cholesterol through statins has been extensively studied over the years and has demonstrated a significant decrease in rates of cardiovascular disease, particularly in high- and very high-risk groups.

Study design

This cross-sectional study enrolled 503 adult patients undergoing lipid-lowering therapy for primary and secondary prevention of ASCVD at the Azadi General & Teaching Hospital in Duhok City, Iraq. Data were collected from January 2, 2023, to October 31, 2023. The sample size was carefully determined to ensure a precise estimation of the primary outcome measure.

Results

Of the 503 patients aged 21-89 years, 315 (62.2%) were women. Among the 145 (28.8%) with ASCVD, 127 (87.5%) had coronary artery disease. Only 150 (29.8%) were on a high-intensity statin, compared to 293 (58.25%) on a moderate-intensity statin. In total, 155 (30.8%) attained LDL-C control (p<0.0001). Among the 207 with very high cardiovascular disease risk, only 10 (4.83%) achieved an LDL-C level below 55 mg/dl.

Conclusion

This study revealed inadequate management of LDL-C levels across various participant categories, particularly those classified as having high cardiovascular disease risk. Control of other risk factors (e.g., hypertension, diabetes, and metabolic syndrome) was overall very poor. Most participants were overweight or obese.

## Introduction

As a leading cause of global mortality, atherosclerotic cardiovascular diseases (ASCVDs) such as acute myocardial infarction and cerebrovascular accidents contribute to 17.3 million deaths annually [1,2]. Without effective intervention, this number is projected to increase to nearly 25 million by 2030 [3]. Research from the Middle East suggests that ASCVD-related deaths are highest in this region of the world [4]. Studies from Saudi Arabia revealed that 22% to 42% of all deaths are linked to cardiovascular diseases [5]. In the Kurdistan region of Iraq, cardiovascular diseases account for 52.6% of all registered deaths [6].

Hyperlipidemia and elevated low-density lipoprotein cholesterol (LDL-C) are major risk factors for ASCVD [7]. Decades of research support the efficacy of lowering LDL-C with statins, making them a cornerstone in treatment, particularly among those at high risk of ASCVD [8-10]. A 1% reduction in LDL-C correlates with a 1% decrease in the risk of ischemic heart disease [11]. Furthermore, a 1% increase in HDL-C is associated with a 3% reduction in the risk of death or myocardial infarction [12].

Statins, either alone or in combination with other drugs like PCSK9 inhibitors, ezetimibe, omega-3, and fibrates, are employed to lower lipid levels [13,14]. The guidelines set by the European Society of Cardiology and European Atherosclerosis Society advocate using statins as the primary intervention for lowering LDL-C levels. The recommended objective is to achieve a minimum 50% reduction from baseline, aiming for LDL-C levels below 70 mg/dl for high-risk individuals and below 55 mg/dl for those classified as very high risk. If treatment with statins proves ineffective in achieving the target, guidelines recommend incorporating Ezetimibe into the regimen, potentially resulting in an additional reduction of LDL-C by 15-20% [15,16]. The utilization of lipid-lowering therapy (LLT) could be linked to certain adverse effects such as increased liver enzymes, hepatitis, muscle discomfort, myopathy, and in extremely rare instances, rhabdomyolysis [16]. However, achieving these targets is challenging. An Austrian study conducted between 2019 and 2020 found that only 5.9% to 38.5% of patients achieved LDL-C levels below 70 mg/dl [17].

Despite robust evidence supporting high-intensity statin use and the importance of dose escalation, real-world reports indicate a gap in adherence to these practices [18,19]. Suboptimal lipid reduction due to insufficient drug doses may increase the risk of future cardiovascular events [20]. In this cross-sectional study, our objective was to assess primary and secondary prevention of ASCVD among patients with dyslipidemia, particularly relating to the use of different intensities of lipid-lowering drugs, management of risk factors, and attainment of LDL-C levels based on joint guidelines from the European Society of Cardiology (ESC) and European Atherosclerosis Society (EAS).

## Materials and methods

Study design

This cross-sectional study enrolled 503 adult patients undergoing lipid-lowering therapy (LLT) at Azadi General Teaching Hospital in Duhok, Iraq, from January 2, 2023, to October 31, 2023. The ethical committee of the College of Medicine of the University of Duhok approved the study on December 15, 2022. The sample size was established to enable accurate computation of the primary outcome metric. Data collection occurred during the patients’ routine hospital visits, thus avoiding the need for any special study-related appointments.

Eligibility criteria

Inclusion criteria of the participants included those aged 18 years or older who were currently taking or had been prescribed LLT within 12 months before enrollment and who had undergone lipid profile measurement within 14 months before enrollment. All participants provided informed consent. Exclusion criteria included individuals with known familial hypercholesterolemia who have a history of cardiovascular disease, any conditions influencing decision-making, human immunodeficiency virus (HIV) and women who were breastfeeding, pregnant, or planning to become pregnant.

Data extraction

During the participation visit, we recorded the patient’s demographic information, height, weight, waist circumference, medical history, and blood pressure, as well as the latest lipid measurements within 14 months before enrollment, any LLT prescription within the previous 12 months, any history of side effects from statins or other drugs, reasons for LLT use, and concomitant medications.

Statistical analyses

Patients’ general and medical characteristics are expressed as a mean (%) or standard deviation (SD). The prevalence rates of cardiovascular diseases among patients with different socio-demographic and medical characteristics are expressed in numbers and percentages. Biomedical measurement comparisons among patients with various cardiovascular diseases were assessed using an independent t-test. The distribution of statin therapy, analyzed by gender and age group, was examined via Pearson chi-squared test. A p-value < 0.05 was considered significant. Statistical calculations were conducted using JMP Pro 14.3.0. (https://www.jmp.com/en_us/home.html).

## Results

Of the 503 enrolled individuals aged 21 to 89 years, 315 (62.2%) were female, 145 (28.8%) were diagnosed with ASCVD, and 127 (87.5%) had coronary artery disease. Diabetes mellitus was present in 292 (58.05%) patients, 284 (97.3%) of whom had type II diabetes mellitus. Among them, 210 (71.92%) had HbA1c levels exceeding the target (>7%), and 397 (78.93%) had uncontrolled blood pressure. Although 453 (90%) participants were either overweight or obese, only 240 (47.71%) reported engaging in physical activity. Moreover, 364 (72.37%) satisfied the criteria for metabolic syndrome, as defined by the International Diabetes Federation. Familial hypercholesterolemia, based on the Simon Broome criteria, was present in 34 (6.7%) patients. Table 1 summarizes the results.

**Table 1:** Participant characteristics (N=503) No. = Number of Patients; % = 100 percentage; ASCVD = Atherosclerotic cardiovascular disease; CKD = Chronic kidney disease; SBP = Systolic blood pressure; DBP = Diastolic blood pressure P < 0.05 considered statistically significant

Characteristics (n=503)		Number	Percentage (%)
History of ASCVD	No	358	71.17
	Yes	145	28.83
ASCVD-coronary	No	376	74.75
	Yes	127	25.25
ASCVD-cerebral	No	465	92.45
	Yes	38	7.55
ASCVD-peripheral	No	458	91.05
	Yes	45	8.95
Sex	Female	315	62.62
	Male	188	37.38
Age groups (Years)	21-29	15	2.98
	30-39	60	11.93
	40-49	124	24.65
	50-59	164	32.60
	60-69	103	20.48
	70-79	34	6.76
	80-89	3	0.60
Smoking	Never smoker	352	69.98
	Ex-smoker	36	7.16
	Heavy smoker	75	14.91
	Light smoker	5	0.99
	Moderate smoker	35	6.96
Familial hypercholesterolemia	No	469	93.24
	Yes	34	6.76
BMI category	Healthy weight	50	9.94
	Obesity Class I	241	47.91
	Obesity Class II-III	40	7.95
	Overweight	172	34.19
Diabetes type	None diabetic	211	41.95
	T1DM	8	1.59
	T2DM	284	56.46
Hypertension	No	130	25.84
	Yes	373	74.16
Microvascular complication (DM)	No	146	50.0
	Yes	146	50.0
Erectile dysfunction	No	100	53.19
	Yes	88	46.81
Sedentary lifestyle	No	240	47.71
	Yes	263	52.29
Family history of premature ASCVD	No	284	56.46
	Yes	219	43.54
Blood pressure	Uncontrolled	397	78.93
	Controlled (SBP <130 and DBP<80)	106	21.07
HbA1c	Controlled HbA1c (<7%)	82	28.08
	Uncontrolled HbA1c	210	71.92
CKD	No	352	69.98
	Yes	151	30.02
Metabolic Syndrome (MetS)	No	139	27.63
	Yes	364	72.37

Regarding cardiovascular risk profiles and LLT, a 10-year risk assessment based on the 2019 ESC/EAS lipid management guidelines was performed for all cases involving primary prevention. Notably, 201 (41.15%) patients were classified as very high risk, 40 (7.95%) as high risk, 96 (19.09%) as moderate, and the remainder as low risk. Only 150 (29.82%) were prescribed high-intensity statins, compared to 293 (58.25%) on moderate-intensity statins. Rosuvastatin (20 mg) was the most commonly prescribed high-intensity statin. Surprisingly, 60 (11.93%) patients were not on any statin treatment. Additionally, 20 (3.98%) patients were using fenofibrate, compared to only 10 (1.99%) receiving ezetimibe at enrollment. No patients were taking PCSK9 inhibitors due to their unavailability in our region. Table 2 summarizes the results.

**Table 2:** Cardiovascular risk patterns, characteristics, and lipid-lowering therapy (N=503) No. = Number of Patient; % = 100 percentage; ESC = European Society of Cardiology; EAS = European Atherosclerosis Society P < 0.05 considered statistically significant

Characteristics (n=503) and Drugs		Number	Percentage
Statins	Not taken	60	11.93
	Atorvastatin 10 mg	6	1.19
	Atorvastatin 20 mg	161	32.01
	Atorvastatin 40 mg	9	1.79
	Rosuvastatin 10 mg	122	24.25
	Rosuvastatin 20 mg	137	27.24
	Rosuvastatin 40 mg	4	0.80
	Simvastatin 20	4	0.80
Statin therapy	Not taken	60	11.93
	High-intensity statin	150	29.82
	Moderate-intensity statin	293	58.25
On statin	Contra-indicated	6	1.19
	Intolerant	15	2.98
	Reluctant	39	7.75
	Yes	443	88.07
Other lipid-lowering drugs	Ezetimibe 10 mg	10	1.99
	Fenofibrate 200 mg	20	3.98
	No	473	94.04
Risk groups according to ESC/EAS	Low	160	31.81
	Intermediate risk	96	19.09
	High risk	40	7.95
	Very high risk	207	41.15

Most individuals in both the high- and very high-risk categories were prescribed moderate-intensity statins (24 [60.00%] and 121 [58.45%], respectively), compared to high-intensity statins (11 [27.50%] and 61 [29.47%], respectively). No statistically significant differences were observed in compliance and statin utilization across various risk groups, as outlined in Table 3.

**Table 3:** Statin therapy according to risk level No. = Number of Patients; % = 100 percentage P < 0.05 considered statistically significant

Statin therapy	Risk no (%)				
	Low (n=160)	Intermediate risk (n=96)	High risk (n=40)	Very high risk (n=207)	p-value (two-sided)
Not taken	16 (10.00)	14 (14.58)	5 (12.50)	25 (12.08)	0.9518
Moderate-intensity statin	96 (60.00)	52 (54.17)	24 (60.00)	121 (58.45)	
High-intensity statin	48 (30.00)	30 (31.25)	11 (27.50)	61 (29.47)	
Statistical analyses were performed by Pearson chi-squared test					

Only 142 (28.23%) achieved LDL-C control, according to 2019 ESC/EAS guidelines, whereas 361 (71.76%) did not (p-value <0.0001). Among 207 patients with very high cardiovascular disease risk, only 10 (4.83%) reached an LDL-C level below 55 mg/dl, with the remaining 197 (95.16%) exceeding that threshold. In the high-risk group of 40 patients, 3 (7.5%) attained an LDL-C level below 70 mg/dl. In the intermediate-risk group of 96 patients, 45 (46.8%) achieved a target LDL below 100 mg/dl. Table 4 summarizes the results.

**Table 4:** LDL-C levels based on 2019 joint guidelines from the European Society of Cardiology (ESC) and European Atherosclerosis Society (EAS) No. = Number of Patients; % = 100 percentage; LDL-C = Low-density lipoprotein cholesterol P < 0.05 considered statistically significant

Risk by ESC		LDL no (%)			p-value	
		Controlled		Uncontrolled		
Low risk		84 (59.15)		76 (21.05)	<0.0001	
Intermediate risk		45 (31.69)		51 (14.12)		
High risk		3 (2.11)		37 (10.24)		
Very high risk		10 (7.04)		197 (54.57)		
Total		142 (28.23)		361 (71.76)		
Statistical analyses were performed by Pearson chi-squared test.						

Comparing results using the previous 2016 ESC/EAS guidelines shows improvement in the overall control rate, that is, from 142 (28.23%), as shown in Table 4, to 176 (34.99%), as shown in Table 5. The improvement is particularly notable in the low and intermediate-risk group, as there is only marginal progress in participants classified as having high or very high cardiovascular disease risk. Among the 207 very high-risk participants, only 23 (11.11%) achieved LDL-C levels below 70 mg/dl. The statistical analysis indicates highly significant results (p-value < 0.0001) for the uncontrolled cases.

**Table 5:** Rate of LDL-C control based on 2016 joint guidelines from the European Society of Cardiology (ESC) and European Atherosclerosis Society (EAS) No. = Number of Patients; % = 100 percentage; LDL-C: Low-density lipoprotein cholesterol P < 0.05 considered statistically significant

Risk by ESC		LDL no (%)			p-value
		Controlled		Uncontrolled	
Low and intermediate risk		141 (80.11)		115 (35.16)	P-value <0.0001
High risk		12 (6.81)		28 (8.56)	
Very high risk		23 (13.06)		184 (56.26)	
Total		176 (34.99)		327 (65)	
Statistical analyses were performed by Pearson chi-squared test.					

The average total LDL among participants was 116.58 mg/dl. For those not on statin therapy, the mean LDL was 148.48 mg/dl, which decreased to 104.09 mg/dl in patients receiving moderate-intensity statins and further dropped to 96.94 mg/dl in the high-intensity statin group (p-value < 0.0001). Table 6 summarizes the results.

**Table 6:** Mean LDL among those on statin therapy No. = Number of Patients; SD = Standard deviation; % = 100 percentage; HSD = Honestly significant difference; LDL-C = Low-density lipoprotein cholesterol P < 0.05 considered statistically significant

Statin therapy		Mean LDL-C mg/dl		SD		P
Not taken		148.48		22.01		<0.0001
Moderate-intensity statin		104.09		31.44		
High-intensity statin		96.94		36.77		
Mean total LDL	116.58					
Pairwise comparisons: Moderate and high-intensity statin therapy > not taken (P<0.0001)						
ANOVA one-way was performed for statistical analyses. The pairwise comparisons were performed using a Turkey HSD test.						

Table 7 summarizes the biomedical measurements. As shown, individuals with confirmed coronary artery disease exhibited notably elevated values for systolic and diastolic blood pressure, diabetes duration, HbA1c levels, waist circumference, as well as a reduced glomerular filtration rate.

**Table 7:** Comparisons of biomedical measurements (N=503) No. = Number of Patients; % = 100 percentage; ASCVD = Atherosclerotic cardiovascular disease; SBP = Systolic blood pressure; DBP = Diastolic blood pressure; WC = Waist circumference; GFR = Glomerular filtration rate; LDL = Low-density lipoprotein; HDL = High-density lipoprotein; SD = Standard deviation P < 0.05 considered statistically significant

Biomedical measurements (n=503)	CVD diseases mean (SD)						
		History of ASCVD					
		No		Yes	Mean diff. (95% CI)		p-value (two-sided)
WC (cm)		97.89 (11.29)		100.50 (12.87)	2.61 (0.33 to 4.90)		0.0249
BMI		31.19 (5.20)		31.32 (4.74)	0.13 (-0.87 to 1.12)		0.8017
SBP		139.70 (23.61)		150.70 (25.83)	11.00 (6.30 to 15.70)		<0.0001
DBP		83.71 (11.71)		88.36 (10.51)	4.65 (2.41 to 6.89)		<0.0001
DM Disease duration		6.12 (4.68)		11.06 (7.43)	4.94 (3.44 to 6.44)		<0.0001
GFR		101.18 (13.85)		82.80 (20.32)	-18.37 (-21.50 to -15.25)		<0.0001
Total cholesterol		182.64 (39.64)		176.69 (43.66)	-5.95 (-13.87 to 1.98)		0.1412
LDL		109.87 (35.87)		104.17 (37.33)	-5.69 (-13.04 to 1.66)		0.1288
HDL		45.82 (10.20)		44.53 (11.18)	-1.29 (-3.34 to 0.75)		0.2141
TG		156.64 (61.52)		153.35 (53.39)	-3.30 (-15.35 to 8.76)		0.5916
LDL/HDL ratio		2.49 (0.99)		2.34 (0.87)	-0.14 (-0.34 to 0.05)		0.1486
HbA1C		8.03 (1.84)		8.95 (2.08)	0.92 (0.46 to 1.39)		0.0001

## Discussion

In this cross-sectional study, only 28% of participants achieved control of LDL-C levels based on the 2019 ESC/EAS guidelines. In comparison, the European DAVINCI study reported a slightly higher rate, with around one-quarter of its participants reaching the LDL-C goal, but this rate declined with increasing cardiovascular disease risk. Specifically, in our study, in the very high-risk group (207 patients), only 4.83% attained the goal (Table 4), which increased to 34.99% overall and 11.11% for the very high-risk group (207 patients) if using the 2016 ESC/EAS guidelines (Table 5). In the European DAVINCI study, 44% achieved the LDL-C goal [21].

Our findings also were lower than those observed in a sub-analysis of the DAVINCI study in Austria, where 58% achieved the 2016 ESC/EAS LDL-C goal [22], and 38% achieved the 2019 ESC/EAS LDL-C goal [11].

Further, our results indicate a lower rate of achievement in comparison to the Centralized pan-Middle East Survey on the under-treatment of hypercholesterolemia (CEPHEUS), a study conducted in six Gulf countries in which 52% of patients reached the LDL-C goal based on the updated guidelines of the National Cholesterol Education Program’s Adult Treatment Panel III. Furthermore, an observational analysis in the United Arab Emirates involving 416 patients with stable coronary artery disease and acute coronary syndrome reported that 39.3% of patients treated with LLT achieved an LDL-C level below 70 mg/dl [23]. The DYSIS-Middle East cross-sectional observational study included 2,182 participants from Saudi Arabia, Lebanon, Jordan, and the United Arab Emirates, 82% of whom were classified as very high-risk and undergoing chronic statin treatment [24]. Overall, LDL-C goal levels were not achieved in 61.8%, among whom 69.5% were very high-risk [24]. In our study, 58% of participants were on moderate-intensity statins, approximately 30% on high-intensity statins, only 1.99% on ezetimibe, and around 12% were not taking any LLT. In contrast, statin use in the DAVINCI study was as follows: 70% were on moderate-intensity statins, 5% on ezetimibe, and 8% were not taking any LLT [22]. In the United Arab Emirates study, 7% were prescribed ezetimibe, and 2.3% were not on any LLT [23]. DYSIS-Middle East reported around 17% receiving ezetimibe, either alone or in combination with statins [24]. Poor statin dose escalation, low ezetimibe prescription rates, and unavailability of PCSK9 inhibitors may contribute to inadequate lipid control [25,26].

Physicians' unfamiliarity with recommendations and guidelines, the high expenses of drugs like PCSK9 inhibitors, patients' reluctance to adopt aggressive LLT, and concerns about statin-related adverse events could all contribute to the suboptimal management of lipid levels [22].

Our study revealed very poor control of other risk factors, with 453 (90%) participants classified as overweight or obese, 292 (58%) having diabetes, and 210 (71.92%) having uncontrolled HbA1c (>7%). Although only 373 (74.16%) were known to be hypertensive, 397 (78.93%) had abnormal blood pressure at enrollment, and 364 (72.37%) met the International Diabetes Federation criteria for metabolic syndrome. In comparison, in a large study from China involving 136,945 participants aged 40-100 years, 64% were overweight or obese, 30% had diabetes, and 62% had hypertension [27]. Our results align with those observed in a Saudi Arabian cross-sectional study of patients with dyslipidemia, where 72.6% were overweight or obese, 71.8% hypertensive, and 59.2% diabetic [28]. Our study's mean LDL-C cholesterol was 116.58 mg/dl, higher than the mean of 97 mg/dl in the DAVINCI European study [21].

Limitations

A limitation of this cross-sectional study is the lack of long-term follow-up with results limited to a single hospital. The absence of similar studies in other centers and hospitals within the region also hinders the generalizability of the results. Nevertheless, it is noteworthy that a substantial proportion of participants in our cohort were either overweight or obese, emphasizing the broader challenge of addressing lifestyle-related risk factors contributing to cardiovascular health issues.

## Conclusions

The conclusions drawn from this study underscore a notable and concerning inadequacy in the management of LDL-C levels across diverse participant cohorts, particularly those at high and very high risk of cardiovascular disease. One prominent observation from the findings is the reliance on monotherapy, primarily statins, indicating a potential limitation in current LLTs. Furthermore, the study sheds light on the suboptimal control of additional cardiovascular risk factors, including hypertension, diabetes, and metabolic syndrome, revealing a significant deficit in the holistic management of cardiovascular disease risk.

It is recommended to implement comprehensive strategies for managing LDL-C levels, especially among individuals at high and very high risk of cardiovascular disease. This should involve moving beyond monotherapy, such as statins, to explore and integrate additional lipid-lowering therapies (LLTs) where appropriate.

## References

[1] Feigin VL (2019). Anthology of stroke epidemiology in the 20th and 21st centuries: assessing the past, the present, and envisioning the future. Int J Stroke.

[2] Benjamin EJ, Blaha MJ, Chiuve SE (2017). Heart Disease and Stroke Statistics-2017 update: a report from the American Heart Association. Circulation.

[3] Mendis S, Lindholm LH, Anderson SG (2011). Total cardiovascular risk approach to improve efficiency of cardiovascular prevention in resource constrain settings. J Clin Epidemiol.

[4] Ramahi TM (2010). Cardiovascular disease in the Asia Middle East region: global trends and local implications. Asia Pac J Public Health.

[5] Mahmood D, Jahan K, Habibullah K (2015). Primary prevention with statins in cardiovascular diseases: a Saudi Arabian perspective. J Saudi Heart Assoc.

[6] Salih SO, Moramarco S, Di Giovanni D, Qadir SA, Alsilefanee HH, Basa FB, Gialloreti LE (2021). Ten-year mortality trends and natural causes of death in the Iraqi Kurdistan. Open Public Health J.

[7] Lee ZV, Llanes EJ, Sukmawan R, Thongtang N, Ho HQ, Barter P (2021). Prevalence of plasma lipid disorders with an emphasis on LDL cholesterol in selected countries in the Asia-Pacific region. Lipids Health Dis.

[8] Fulcher J, O'Connell R, Voysey M (2015). Efficacy and safety of LDL-lowering therapy among men and women: meta-analysis of individual data from 174,000 participants in 27 randomised trials. Lancet.

[9] Schwartz GG, Fayyad R, Szarek M, DeMicco D, Olsson AG (2017). Early, intensive statin treatment reduces 'hard' cardiovascular outcomes after acute coronary syndrome. Eur J Prev Cardiol.

[10] LaRosa JC, Grundy SM, Waters DD (2005). Intensive lipid lowering with atorvastatin in patients with stable coronary disease. N Engl J Med.

[11] Grundy SM, Cleeman JI, Merz CN (2004). Implications of recent clinical trials for the National Cholesterol Education Program Adult Treatment Panel III guidelines. Circulation.

[12] Boden WE (2000). High-density lipoprotein cholesterol as an independent risk factor in cardiovascular disease: assessing the data from Framingham to the Veterans Affairs High--Density Lipoprotein Intervention Trial. Am J Cardiol.

[13] Choi HD, Chae SM (2018). Comparison of efficacy and safety of combination therapy with statins and omega-3 fatty acids versus statin monotherapy in patients with dyslipidemia: a systematic review and meta-analysis. Medicine (Baltimore).

[14] Gallego-Colon E, Daum A, Yosefy C (2020). Statins and PCSK9 inhibitors: a new lipid-lowering therapy. Eur J Pharmacol.

[15] Catapano AL, Graham I, De Backer G (2016). 2016 ESC/EAS guidelines for the management of dyslipidaemias. Eur Heart J.

[16] Mach F, Baigent C, Catapano AL (2020). 2019 ESC/EAS Guidelines for the management of dyslipidaemias: lipid modification to reduce cardiovascular risk: The Task Force for the management of dyslipidaemias of the European Society of Cardiology (ESC) and European Atherosclerosis Society (EAS). Eur Heart J.

[17] Pichler M, Lautsch D, Adler C (2013). Are there differences in LDL-C target value attainment in Austrian federal states? Yes!. Wien Med Wochenschr.

[18] Banefelt J, Lindh M, Svensson MK, Eliasson B, Tai MH (2020). Statin dose titration patterns and subsequent major cardiovascular events in very high-risk patients: estimates from Swedish population-based registry data. Eur Heart J Qual Care Clin Outcomes.

[19] Lee SH, Song WH, Jeong MH (2019). Dyslipidemia and rate of under-target low-density lipoprotein-cholesterol in patients with coronary artery disease in Korea. J Lipid Atheroscler.

[20] Akyea RK, Kai J, Qureshi N, Iyen B, Weng SF (2019). Sub-optimal cholesterol response to initiation of statins and future risk of cardiovascular disease. Heart.

[21] Ray KK, Molemans B, Schoonen WM (2021). EU-wide cross-sectional observational study of lipid-modifying therapy use in secondary and primary care: the DA VINCI study. Eur J Prev Cardiol.

[22] Siostrzonek P, Brath H, Zweiker R, Drexel H, Hoelzl R, Hemetsberger M, Ray KK (2022). Lipid lowering therapy in primary and secondary prevention in Austria: are LDL-C goals achieved?: Results from the DA VINCI study. Wien Klin Wochenschr.

[23] Al Mahmeed W, Bakir S, Beshyah SA (2019). Prevalence of lipid abnormalities and cholesterol target value attainment in patients with stable and acute coronary heart disease in the United Arab Emirates. Heart Views.

[24] Al Sifri SN, Almahmeed W, Azar S (2014). Results of the Dyslipidemia International Study (DYSIS)-Middle East: clinical perspective on the prevalence and characteristics of lipid abnormalities in the setting of chronic statin treatment. PLoS One.

[25] Banach M, Penson PE (2020). Statins and LDL-C in secondary prevention-So much progress, so far to go. JAMA Netw Open.

[26] Banach M, Penson PE (2021). Lipid-lowering therapies: better together. Atherosclerosis.

[27] Opoku S, Gan Y, Fu W (2019). Prevalence and risk factors for dyslipidemia among adults in rural and urban China: findings from the China National Stroke Screening and prevention project (CNSSPP). BMC Public Health.

[28] Alzahrani GS, Aljehani SM, Al-Johani JJ (2018). Risk factors of dyslipidemia among Saudi population 2017. Egypt J Hosp Medicine.

